# Introduction to celebrating recent chemical science in Mexico

**DOI:** 10.1039/d0ra90134b

**Published:** 2021-01-11

**Authors:** Gabriel Merino, María de Jesús Rosales, Alberto Vela

**Affiliations:** Department of Applied Physics, Cinvestav Mexico gmerino@cinvestav.mx; Department of Chemistry, Cinvestav Mexico

## Abstract

Gabriel Merino, María de Jesús Rosales and Alberto Vela introduce the *PCCP*, *Dalton Transactions*, *New Journal of Chemistry* and *RSC Advances* themed issue on celebrating recent chemical science in Mexico.
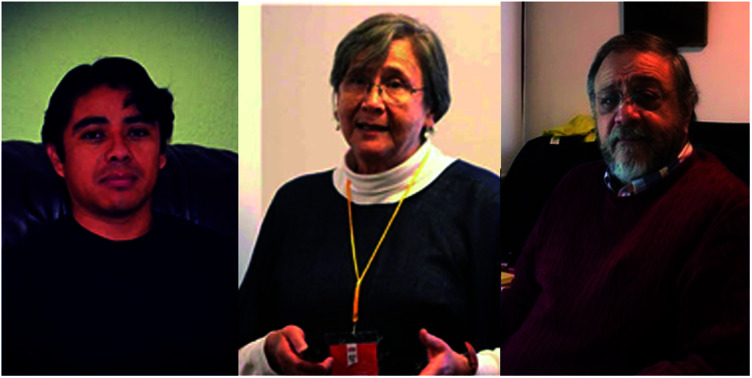

Chemical research in Mexico has a long tradition. It dates back to the establishment of several mining companies by the Spaniards in the 16th and 18th centuries. In the middle of the last century, this area excelled mainly in natural products and organic chemistry due to the discovery of sex hormone precursors in some Mexican plants. That era witnessed the creation of various research centres, including the Institute of Chemistry at Universidad Nacional Autónoma de México (the largest public university in the country) and the Department of Chemistry at Centro de Investigación y de Estudios Avanzados (an institution founded in the 1960s with strong ties to the National Polytechnic Institute). The dominant research areas at both institutions were organic chemistry and natural products. After a few years, the vision and enthusiasm of several Mexican chemists and authorities at these institutions made the diversification of chemical research in Mexico possible. By the ‘70s, groups started to work in inorganic chemistry, analytical chemistry, physical chemistry, biochemistry, radiochemistry, and theoretical chemistry. Also, new higher education and research centers were created like UAM (Universidad Autónoma Metropolitana) and IMP (Instituto Mexicano del Petróleo). Over the last 30 years, Mexican chemists have worked hard to establish their reputation within the global research community. This progress is reflected in the number of papers published in high-ranking journals of related fields. While other areas are making progress, the number of papers published by Mexican scientists in organic chemistry, physical chemistry, and inorganic chemistry is remarkable. Similar to international trends, supramolecular chemistry, materials chemistry, and nanochemistry have become areas of importance in Mexico.

The publications in this revision were selected from 250 articles in *PCCP*, *Dalton Transactions*, *RSC Advances*, and *New Journal of Chemistry* with at least one author having an address in Mexico over the period 2018–2020. The following criteria were applied: (1) the corresponding author must belong to a Mexican institution, public or private, and (2) in cases of corresponding authors with multiple articles, only the most representative publication was considered. We consider that this blind selection (72 contributions) resulted in a fair and representative sample of the chemistry that is currently being done in Mexico, including the most important Mexican institutions where this chemical research is taking place. Remarkably, a significant number of the corresponding authors in this compilation are young researchers, many of whom studied abroad or made at least one postdoctoral stay outside Mexico. These young scientists have been hired by both the long-standing institutions and universities and research centres outside Mexico City. The new generation of Mexican chemical researchers has certainly arrived, aiming to contribute with knowledge at the highest level.

Not everything looks promising on the road ahead. Access to major equipment, easy and expeditious access to chemicals, and a declining interest in chemistry are some of the main obstacles to strengthening chemical research in Mexico. In the same vein, the financial support for science in Mexico has decreased substantially. The financial avenues established over the years to acquire the necessary materials for year-round research projects are being dismantled.^1,2^ Calls for funding from the federal government have been severely reduced and the financial mechanisms to facilitate the continuity of research are being scrutinized, with a very high risk of disappearing.^1–4^ The immediate future of science in Mexico does not look too promising. Despite these unfortunate situations, it is highly stimulating to see that chemists in Mexico are active and productive, contributing in some of the best scientific journals in the discipline, like the journals published by the Royal Society of Chemistry. We hope that the changes currently underway in the country, together with the enthusiasm and the new ideas of the Mexican chemical community, will continue contributing with even more and better scientific works to the chemical sciences.

## Supplementary Material

## References

[cit1] Lazcano A. (2019). Quo vadis, Mexican science?. Science.

[cit2] Álvarez-Buylla Roces M. E. (2019). A new scientific agenda for Mexico. Science.

[cit3] Guglielmi G. (2019). Mexican science suffers under budget cuts. Nature.

[cit4] Mega E. R. (2019). Mexican science faces its #MeToo moment. Nature.

